# Supplementary material for: Cyclin-dependent kinase control of motile ciliogenesis
Source: eLife. 2018 Aug 28;7:e36375. doi: 10.7554/eLife.36375 (PMC6145839; doi:10.7554/eLife.36375)
Supplement: Supplementary file 1. — Supplementary Table 2 Quantitative realtime PCR primers used. All primers amplify the mouse target unless otherwise specified. Supplementary Table 3 Statistical test and replicate information for data. [file elife-36375-supp1.docx]

**Supplementary tables**

Supplementary Table 1. **Antibodies used.**

| **Protein** | **Antibody** | **Source** | **Dilution** | **Fixation*** |
| --- | --- | --- | --- | --- |
| **ac. α-Tubulin** | 6-11b-1 | Abcam | 1 to 2,000 | M or P |
| **α-Tubulin** | DM1a | Abcam | 1 to 10,000 | - |
| **Ccna1** | H230 | Santa Cruz Biotech | 1 to 50 | P |
| **Cdk2** | D12 | Santa Cruz Biotech | 1 to 500 | - |
| **Cdk2 pT160** | 2561 | Cell Signaling | 1 to 500 | - |
| **Foxj1** | 2A5 | eBioscience | 1 to 500 | P or P/M |
| **TRRAP** | HPA038203 | Sigma Aldrich | 1 to 200 | M or P/M |
| **Myb** | D7 | Santa Cruz Biotech | 1 to 500 | P |
| **Pericentrin** | 611814 | BD Biosciences | 1 to 200 | M or P |
| **γ-Tubulin** | GTU88 | Sigma Aldrich | 1 to 1,000 | M |
| **Odf2** | HPA001874 | Sigma Aldrich | 1 to 500 | M |
| **Deup1** | HPA010986 | Sigma Aldrich | 1 to 500 | M |
| **Plk4** | - | Hatch *et al*., 2010 | 1 to 250 | M |
| **Sass6** | 91.390.21 | Santa Cruz Biotech | 1 to 250 | M |
| **Ccp110** | 12780-1-AP | Proteintech | 1 to 500 | M |
| **Cep164** | - | Lau *et al*., 2012 | 1 to 500 | M |
| **E-cadherin** | 13-1900 | Thermo Fisher | 1 to 250 | M or P |
| **GFP** | 4745-1051 | AbD Serotech | 1 to 500 | M or P |
| **HA** | 3F10 | Sigma Aldrich | 1 to 250 | P |
| **myc** | A14 | Santa Cruz Biotech | 1 to 250 | M or P |
|  |  |  |  |  |
| **Fixation conditions used** | |  |  |  |
| M = -20 °C methanol for 10 min at -20 °C | | |  |  |
| P = 4% paraformaldehyde for 10 min at room temperature | | | |  |
| P/M = 0.5% paraformaldehyde for 5 min at room temperature followed by -20C methanol for 10 min at -20C | | | | |

Supplementary Table 2. **Quantitative realtime PCR primers used.** All primers amplify the mouse target unless otherwise specified.

| **Target** | **Primers** | |
| --- | --- | --- |
| ***Ccna1*** (mouse) | TGATGCTTGTCAAATGCTCAGC | AGGTCCTCCTGTACTGCTCAT |
| ***CCNA1*** (human) | GGAAGGCATTTTCTGATCCA | GCTAGGGCTGCTAACTGCAA |
| ***Ccna2*** | GCCTTCACCATTCATGTGGAT | TTGCTGCGGGTAAAGAGACAG |
| ***Ccne1*** | GTGGCTCCGACCTTTCAGTC | CACAGTCTTGTCAATCTTGGCA |
| ***Ccne2*** | AGCCGTTTACAAGCTAAGCAA | TGGCCTGAATTATCTGGGTTTC |
| ***Cdk1*** | AGAAGGTACTTACGGTGTGGT | GAGAGATTTCCCGAATTGCAGT |
| ***Cdk2*** | CCTGCTTATCAATGCAGAGGG | TGCGGGTCACCATTTCAGC |
| ***Mcidas*** | CCTAGTGGTGATTCGTCCGCGTCG | GGTTATTCTCTATGAGAGCAGTC |
| ***Foxj1*** (mouse) | GGCCACCAAGATCACTCTGT | TGTTCAAGGACAGGTTGTGG |
| ***FOXJ1*** (human) | CGAGGCACTTTGATGAAGC | CAACTTCTGCTACTTCCGCC |
| ***Myb*** | CGTCATCTGGTCCTCTGTCTT | TGTCCTCAAAGCCTTTACCG |
| ***Gmnc*** | TGGTCTCCTGGACAACACTG | TAACTCAGAGGGCGATTCCA |
| ***Plk4*** | AGGAGAAACTAATGAGCACCACA | TGGCTCTCGTGTCAGTCCAA |
| ***Sass6*** | ATTCCTTTACGCGGACTTAGC | AAGTAGGCTGAAGACGAGGAG |
| ***Ccdc67*** | ATTACCAAACTCAGCTAAACGGG | ACCAGGCGAGGGATCTCAG |
| ***Cetn2*** (mouse) | ACAGGGCAGAACAAGAGCAC | CCACTGCTTATGGTGACATGG |
| ***CETN2*** (human) | AGCTCAGGCTTAGGGCTCAT | TGTACACGTCGGTTGCCTAA |
| ***Gapdh*** (mouse) | GACTTCAACAGCAACTCCCAC | TCCACCACCCTGTTGCTGTA |
| ***GAPDH*** (human) | TGCACCACCAACTGCTTAGC | GGCATGGACTGTGGTCATGAG |

Supplementary Table 3. **Statistical test and replicate information for data.**

| **Figure** | **Panel** | **Statistics** | **Sample Size and Replicates (*technical replicate*; biological replicate)** |
| --- | --- | --- | --- |
| 1 | A | NA | *n=2* filters were labeled from n=5 distinct culture sets |
| 1 | B | One-way ANOVA, then Bonferroni's multiple comparisons test | n>300 cells were counted in *n=10* areas from n=1 filter from n>3 treatments (n=5 for NU6140, n=3 for other Cdkis) |
| 1 | C | One-way ANOVA, then Bonferroni's multiple comparisons test | expression levels were established from *n=3 duplicate* reactions from n=3 treatments |
| 1 | D | NA | *n=2* filters were labeled from n=3 distinct culture sets |
| 1 | E | One-way ANOVA, then Bonferroni's multiple comparisons test | n>300 cells were counted in *n=10* areas from n=3 treatments |
| 2 | A | NA | *n=2* filters were labeled from n>3 treatments (n=5 for NU6140, n=3 for other drugs) |
| 2 | B | Two tailed paired t test | n>100 GFP+ or myc+ cells were counted from *n=1* treatments from n=3 distinct culture sets |
| 3 | A | NA | *n=2* filters were labeled from n=3 distinct culture sets |
| 3 | B | One-way ANOVA, then Bonferroni's multiple comparisons test | n>100 cells were counted in *n=10* areas from n=3 treatments |
| 3 | C | One-way ANOVA, then Bonferroni's multiple comparisons test | n>100 cells were counted in *n=10* areas from n=3 treatments |
| 3 | D | One-way ANOVA, then Bonferroni's multiple comparisons test | expression levels were established from *n=3 duplicate* reactions from n=3 treatments |
| 4 | A | NA | *n=2* filters were labeled from n=3 distinct culture sets |
| 4 | B | Two tailed paired t test | n=10 cells were quantitated from n=1 experiments |
| 4 | C | NA | *n=2* western blots were generated from n=2 distinct culture timecourses |
| 4 | D | NA | *n=2* filters were labeled from n=3 distinct culture sets |
| 5 | A | One-way ANOVA, then Bonferroni's multiple comparisons test | expression levels were established from *n=3 duplicate* reactions from n=3 timecourses |
| 5 | B | NA | *n=2* western blots were generated from n=2 distinct culture timecourses, testis lysate was prepared only one time |
| 5 | C | NA | expression levels were established from *n=1 duplicate* reactions from n=2 sorts |
| 5 | D | One-way ANOVA, then Bonferroni's multiple comparisons test | expression levels were established from *n=3 duplicate* reactions from n=2 assays |
| 5 | E | One-way ANOVA, then Bonferroni's multiple comparisons test | expression levels were established from *n=3 duplicate* reactions from n=2 assays |
| 6 | A | Two-way ANOVA, then Bonferroni's multiple comparisons test | n>100 GFP+ cells were counted from *n=3* treatments from n=3 distinct culture sets |
| 6 | B | NA | *n=2* filters were labeled from n=3 distinct culture sets |
| 6 | C | NA | *n=2* filters were labeled from n=3 distinct culture sets |
| 6 | D | NA | *n=2* filters were labeled from n=3 distinct culture sets |
| 7 | A | NA | *n=10* areas were analyzed from n=5 wild-type and mutant mice |
| 7 | B | NA | *n=10* areas were analyzed from n=2 wild-type and mutant mice |
| 7 | C | NA | NA |
| S1 | A | NA | NA |
| S1 | B | NA | NA |
| S2 |  | NA | *n=2* filters were labeled from n=3 distinct culture sets |
| S3 |  | NA | *n=2* filters were labeled from n=3 distinct culture sets |
| S4 |  | One-way ANOVA, then Bonferroni's multiple comparisons test | expression levels were established from *n>3 duplicate* reactions from n=3 timecourses |
| S5 | A | NA | expression levels were established from *n=1 duplicate* reactions from n=2 sorts |
| S5 | B | NA | NA |
| S6 | A | NA | *n=2* filters were labeled from n=3 distinct culture sets |
| S6 | B | NA | *n=1* western blots were generated from n=2 cultures |
| S6 | C | NA | *n=2* coverslips were labeled from n=2 distinct culture sets |
| S6 | D | One-way ANOVA, then Bonferroni's multiple comparisons test | expression levels were established from *n=3 duplicate* reactions from n=3 treatments |
| S7 | A | Two tailed paired t test | n>400 cells were counted in *n=1* area from n=3 mice |
| S7 | B | Two-way ANOVA, then Bonferroni's multiple comparisons test | n>100 cells were counted in *n=10* areas from n=3 treatments |
